# Causal role of immune cells on cervical cancer onset revealed by two-sample Mendelian randomization study

**DOI:** 10.1038/s41598-024-65957-7

**Published:** 2024-06-28

**Authors:** Zicheng Zhao, Pengxian Yan, Xiaoyu Zhang, Xiaomin Yu, Fengchun Lv, Mingyu Gong, Xiu-An Yang

**Affiliations:** 1 Laboratory of Genetic Engineering and Genomics, School of Basic Medical Sciences, Chengde Medical University, Chengde, 067000 Hebei China; 2 Graduate School of Chengde Medical University, Chengde, 067000 China; 3 Hebei Key Laboratory of Nerve Injury and Repair, Chengde Medical University, Chengde, 067000 China

**Keywords:** Cervical cancer, Cervical non-neoplastic conditions, Immune traits, Two-sample Mendelian randomization analysis, Causal effects, Tumour immunology, Cervical cancer, Computational biology and bioinformatics

## Abstract

Cervical cancer (CC) is a prevalent gynecological cancer worldwide that significantly impacts the quality of life and the physical and mental well-being of women. However, there have been limited studies utilizing Mendelian randomization (MR) analysis to investigate the connection between immune cells and CC. This study is to investigate the causal effects of immune traits on CC and non-neoplastic conditions of the cervix. The GWAS data for 731 immunophenotypes and six GWAS data for CC from the FinnGen database were downloaded. Subsequently, a two-sample MR analysis was conducted using the MR Egger, Weighted median, Inverse variance weighted (IVW), Simple mode, and Weighted mode methods. Our study has identified the potential causal effects of immune traits on inflammatory diseases of the cervix, other noninflammatory disorders of the cervix uteri, carcinoma in situ of cervix uteri, adenocarcinomas of cervix, squamous cell neoplasms and carcinoma of cervix, as well as malignant neoplasm of the cervix uteri, with the respective numbers being 8, 6, 11, 8, 23, and 12, respectively. A strong correlation between classic monocytes and various cervical diseases was revealed. Furthermore, we discovered that B cells expressing BAFF-R have the ability to impede the advancement of malignant CC, specifically squamous cell neoplasms and carcinoma of cervix. Our study has demonstrated a significant association between immune traits and both CC and non-neoplastic conditions of the cervix through two-sample Mendelian randomization, providing valuable insights for future clinical research.

## Introduction

Cervical cancer (CC) is widely recognized as a significant public health issue and holds the position of being the fourth deadliest form of cancer among women across the globe^1,2^. According to WHO data, approximately 604,000 new cases were diagnosed globally in 2020^3^. CC, especially in developing countries, is identified as the primary cause of death for women worldwide, as stated by the International Agency for Research on Cancer^2^. While most women with early-stage tumors can receive treatment, many experience long-term complications. Chemoradiation (CAT) is the recommended treatment for locally advanced CC^4,5^. The timing of CAT plays a crucial role in determining survival outcomes, and its efficacy in underdeveloped nations remains uncertain due to the absence of suitable screening techniques^6,7^.

Numerous factors, such as smoking, a compromised immune system, and prolonged use of oral contraceptives, contribute to the development of CC^8^. The primary cause of CC is a persistent infection with high-risk types of the human papillomavirus (HPV), particularly HPV16 and/or HPV18, which are the most common culprits^9^. Therefore, further exploration of these HPV types may uncover causes that are linked either directly or indirectly to other malignancies in the human reproductive system. It is well-documented that the tumor microenvironment (TME) plays crucial roles in the occurrence and progression of tumors. In the case of CC, both innate and acquired immune responses are present in HPV-infected lesions^10^. For instance, cell migration to the squamous epidermis, particularly T lymphocytes and Langerhans cells, occurs in response to high-risk HPV infection^11^. The regression or progression of CC depends on the interaction of various cellular immune responses^12^.

Mendelian randomization (MR) is an analytical method that infers the causal effects of exposure on outcomes using genetic variations in non-experimental data. Since alleles are randomly assigned during meiosis, MR can minimize traditional confounding variables and reverse causality, thereby providing stronger evidence for causal inference^13^. MR utilizes existing GWAS data to address some limitations and issues encountered in traditional observational studies and randomized controlled trials. When using MR as a research method, three assumptions must be met: (1) genetic variation must be associated with the exposure factor; (2) genetic variation must affect the outcome through exposure, rather than through other factors; (3) genetic variation is not associated with any known or unknown confounding factors related to the outcome. In general, these three key assumptions must be satisfied to ensure the validity of the analysis. Violating any one of them will introduce bias to any causal inference^13,14^. In conclusion, although MR cannot entirely confirm causal relationships, it can to a large extent provide evidence for causal relationships. The development of CC is hindered by immune cells, and within the TME, there are specific subsets of immune cells that "suppress" tumor immunity, leading to a more intricate tumor microenvironment for CC^15,16^. Currently, there is a lack of extensive research samples that investigate the link between immune inflammation and CC. In this study, a comprehensive two-sample MR analysis was conducted to determine the causal relationship between immune cell characteristics and different types of CC and cervical non-neoplastic conditions (inflammatory diseases of the cervix and other noninflammatory disorders of the cervix uteri).

## Methods

### Genome-wide association study (GWAS) data sources of 731 immune traits

Publicly available GWAS data of 731 immunophenotypes were downloaded from GWAS Catalog (accession number: GCST0001391-GCST0002121) as described previously^17,18^. Briefly, these 731 types of immune traits are divided into four main categories, namely absolute cell (n = 118), relative cell counts (n = 192), morphological parameters (n = 32), and median fluorescence intensities (n = 389). This dataset contains the detailed information of approximately 22 million SNPs genotyped using high-density arrays in 3,757 European individuals, as recorded by Sidore et al.^19^. Detailed information of the immune traits was described previously^19,20^.

### GWAS data sources of various types of CC and cervical non-neoplastic conditions

The GWAS statistical data of various types of CC and cervical non-neoplastic conditions used in this study were obtained from FinnGen Research (https://risteys.finregistry.fi/), including data on inflammatory diseases of the cervix (N14_INFCERVIX, 2009 cases and 205,362 controls), other noninflammatory disorders of cervix uteri (N14_OTHNONINFCERVIX, 1512 cases and 111,583 controls), carcinoma in situ of cervix uteri [CD2_INSITU_CERVIX_UTERI_EXALLC, cervical intraepithelial neoplasia (CIN) III, 2458 cases and 180,963 controls], adenocarcinomas of cervix (C3_CERVIX_ADENO_EXALLC, 116 cases and 182,927 controls), squamous cell neoplasms and carcinoma of cervix (SCNCC, C3_CERVIX_SQUAM_EXALLC, 168 cases and 182,927 controls), and malignant neoplasm of cervix uteri (C3_CERVIX_UTERI_EXALLC, 388 cases and 182,927 controls). Samples starting with C3 represent malignant samples, those starting with CD2 represent benign in situ carcinoma, and N14 represent non-cancerous samples. These six data contained a total of 687,486 samples. The detailed information of GWAS data for CC and cervical non-neoplastic conditions used in this study is shown in Supplementary Tables 1 and 2. Each dataset was initially used as outcome data for the two-sample Mendelian randomization analysis and used as exposures during reverse two-sample MR analysis. After quality control and imputation, approximately 12,132 million variants were analyzed, identifying a total of 1,066 independent single nucleotide polymorphisms (SNPs).

### Instrumental variables (IV) selection

The significance level of each IV related to immune traits was set to 1 × 10^–5^ according to the common standards^3,17,18^. In order to ensure the reliability of the results and prevent bias caused by strong linkage disequilibrium (LD) between SNPs, a threshold of r^2^ < 0.001 was implemented to account for LD effects, with LD regions limited to 10,000 kb^18,21^. To evaluate the strength of each IV and avoid weak instrument bias, the proportion of phenotypic variance explained (PVE) and the F-statistic were calculated. IVs with an F-statistic below 10 were excluded, while those remaining were designated as strong IVs for further analysis.

### Two-sample MR analysis

To investigate the causal relationship between immunophenotypes and the onset of CC, we utilized the TwoSampleMR R software package (version 0.5.8) to conduct two-sample MR analysis in the R software (https://www.r-project.org/, version 4.2.3). MR Egger^22^, Weighted median^23^, Inverse variance weighted (IVW)^24^, Simple mode, and Weighted mode were used for two-sample MR analysis. A positive overall result is determined when the IVW method, along with at least one of the MR Egger, Weighted Median, Simple Mode, or Weighted Mode methods, yield positive outcomes for each analysis. In order to minimize the impact of CC on immune traits, a reverse MR analysis was performed on immunophenotypes that demonstrate a strong association with CC or cervical non-neoplastic conditions.

### Statistical analysis

In the random effects model, IVW was used as the main analytical method. The MR results were derived from the SNPs that were screened below the genome-wide significance threshold (p < 5 × 10^−8^). After obtaining the MR results, sensitivity analyses were conducted, such as heterogeneity testing and multiple-effect testing using the MR Egger method and MR-PRESSO test. The MR Egger method is utilized for the initial identification of horizontal pleiotropy^22^. If the p-value is below 0.05, it signifies the presence of horizontal pleiotropy. The MR-PRESSO outlier test assesses the existence of specific horizontal pleiotropy outlier IVs by contrasting the observed and expected distribution of the test IVs^25^. In comparison to MR Egger, the MR-PRESSO test can more precisely detect biased SNPs^25^. If the p-value is less than 0.05, indicating the presence of a biased SNP, it should be excluded from the IVs; if the p-value is above 0.05, it suggests the absence of a biased SNP. For sensitivity analysis and calculating the remaining cumulative effects of the SNPs, the leave-one-out approach was utilized. To assess if IVs influenced the outcomes through other pathways than exposure, horizontal pleiotropy tests were conducted using the intercept terms derived from the MR-Egger regression^25^. The risk of CC and cervical non-neoplastic conditions in relation to the immune traits was summarized using odds ratios (ORs) and 95% confidence intervals (95% CIs). Subsequently, a criterion of p-values less than 0.05 was used to screen for immune traits associated with CC. Considering reverse MR analysis, results with a p-value of higher than 0.05 were kept.

## Results

### Causal effect of immune traits on cervical non-neoplastic conditions

**Figure 1:**
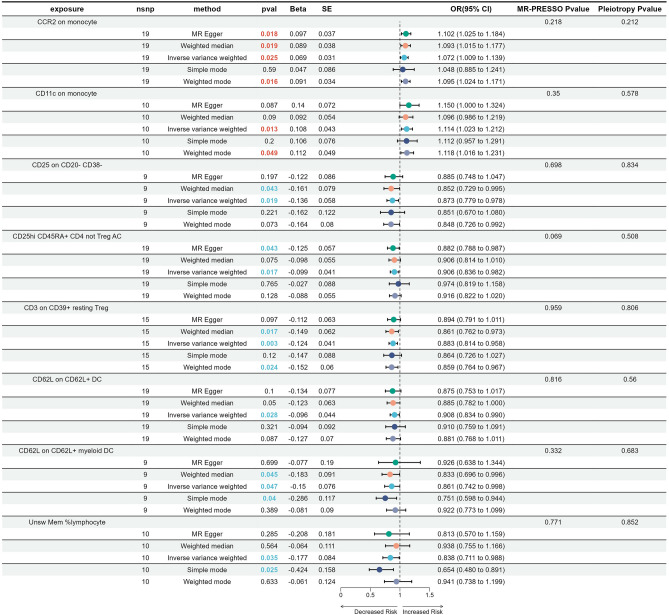
Forest plots showed the causal associations between immune cell traits and inflammatory diseases of the cervix.

**Figure 2:**
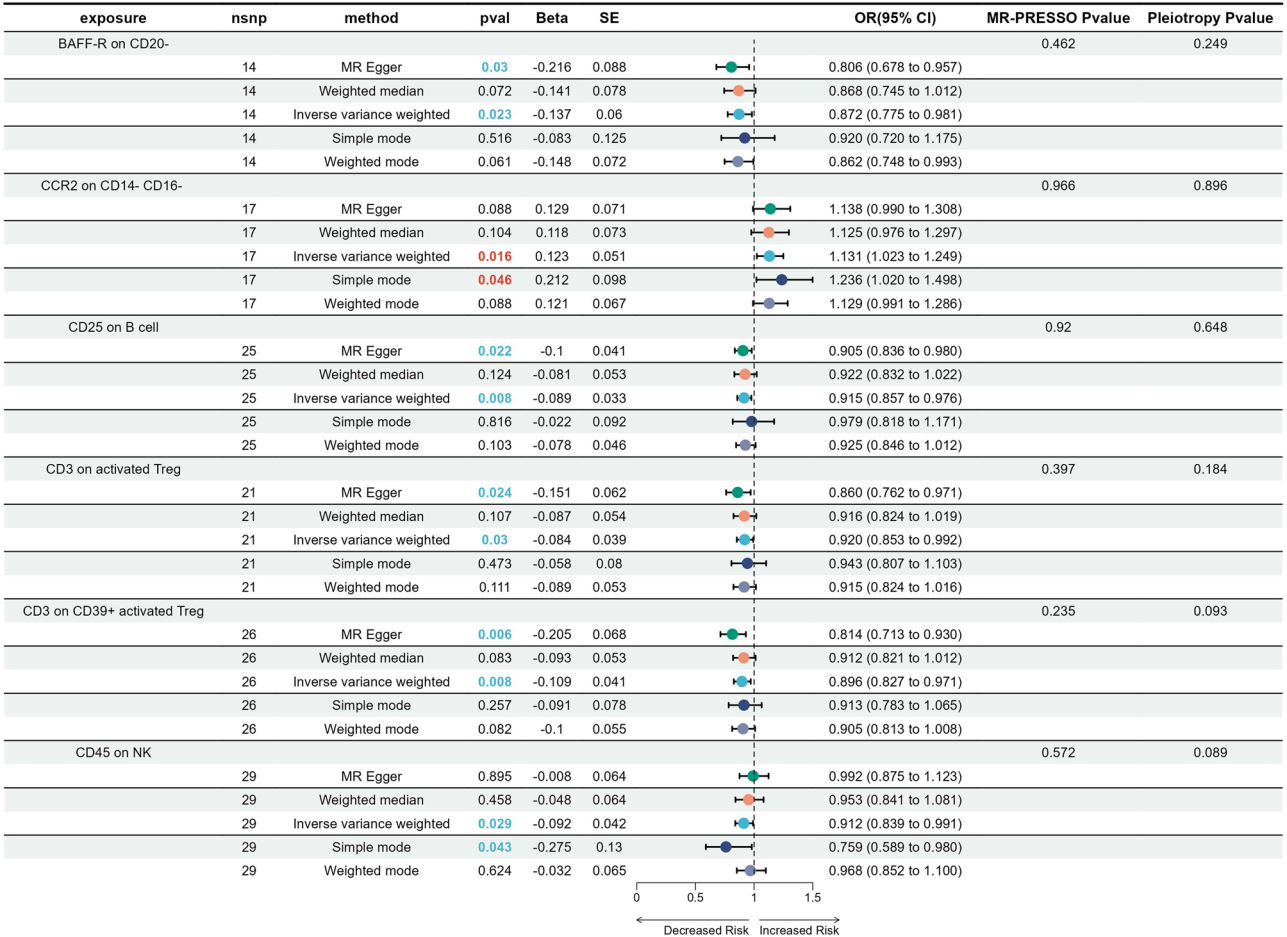
Forest plot to visualize the causal effects of immune cell traits and other noninflammatory disorders of cervix uteri.

To investigate the potential causal effect of immunophenotypes on the onset of CC, a two-sample MR analysis was conducted using GWAS data from 731 immune cells and six datasets comprising different statuses of CC and non-neoplastic samples. We first conducted analyses on two non-neoplastic conditions, namely inflammatory diseases of the cervix and other noninflammatory disorders of the cervix uteri. Following the filtering process, two traits (CCR2 on monocytes and CD11c on monocytes) demonstrated a positive association with inflammatory diseases of the cervix (Fig. 1). Meanwhile, six immune traits [CD3 on CD39+ resting Treg, CD25 on CD20−CD38− (B cell), CD25hi CD45RA+ CD4 not Treg AC, CD62L on CD62L+ DC, CD62L on CD62L+ myeloid DC, and Unsw Mem %lymphocyte (B cell)] displayed potential protective effects against inflammatory diseases of the cervix. The results of MR Egger, Weighted median, IVW, Simple mode, and Weighted mode tests showed a consistent direction, indicating the reliability of the results. Supplementary Fig. 1 illustrated the scatter plots displaying the impact of immune traits on inflammatory diseases of the cervix. In order to ensure the reliability of our results, reverse MR analysis was conducted to examine potential reverse causation using immune traits that displaying a strong association with inflammatory diseases of the cervix. The results showed that there was no evidence of a causal connection between inflammatory diseases of the cervix and immune traits. To summarize, our study identified a causal relationship between specific immunophenotypes and the incidence of inflammatory diseases of the cervix. For the data on other noninflammatory disorders of the cervix uteri, it was found that CCR2 on CD14−CD16− (monocyte) was nominally associated with an increased risk of these disorders. However, BAFF-R on CD20- (B cell), CD3 on activated Treg, CD3 on CD39+ activated Treg, CD25 on B cell, and CD45 on NK showed significant protective effects against cervix disorders (Fig. 2). The scatter plots indicated the stability of the results in Supplementary Fig. 2. Reverse MR analysis did not reveal any evidence of a causal connection between other noninflammatory disorders of the cervix uteri and immune traits. In conclusion, monocytes have been found to have a positive causal correlation with non-neoplastic diseases of the cervix, such as inflammatory conditions and other disorders of the cervix uteri.

### Causal effect of immune traits on carcinoma in situ of cervix uteri

**Figure 3:**
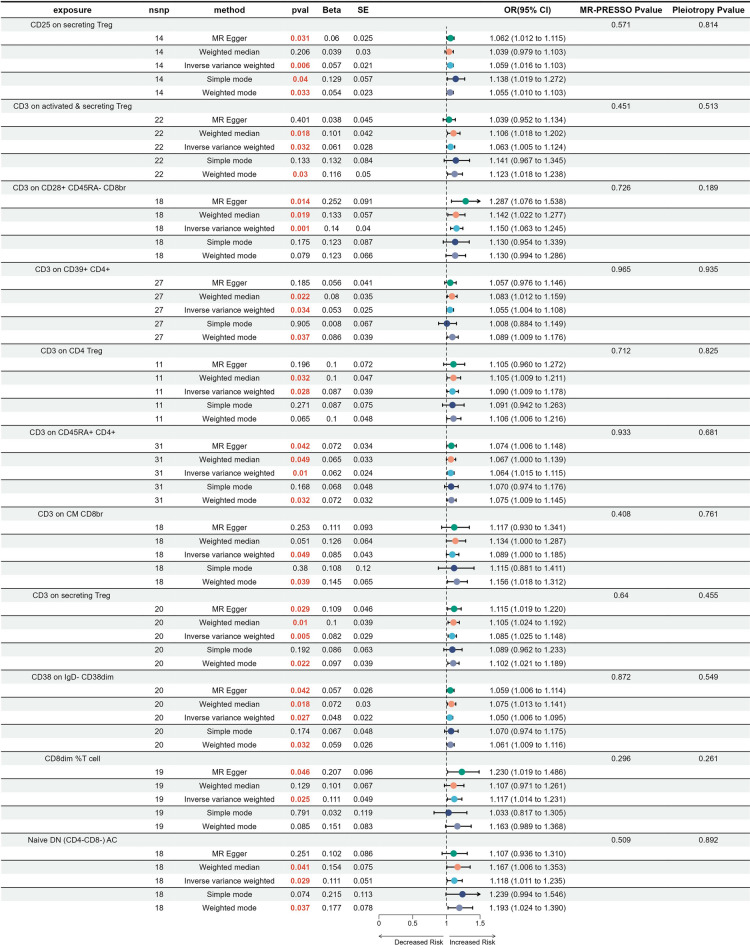
Causal effects to display the immune cell traits and carcinoma in situ of cervix uteri illustrated by forest plot.

We then conducted an analysis of the causal effect of immune traits on carcinoma in situ of the cervix uteri. Finally, 12 types of four major categories of immune cells were found to be related to the in situ GWAS data (Fig. 3). Apart from CD38 on IgD- CD38dim and HLA DR on B cell were B cell lymphocyte, the remaining cells were all different types of T cells. Among these 12 types of immune cells, one type (HLA DR on B cells) exhibited a negative causal estimate towards in situ of cervix uteri, with IVW OR 0.911 (0.833 to 0.996, 0.041). On the other hand, the remaining types (CD25 on secreting Treg, CD3 on activated & secreting Treg, CD3 on CD28+ CD45RA−CD8br, CD3 on CD39+ CD4+, CD3 on CD4 Treg, CD3 on CD45RA+ CD4+, CD3 on CM CD8br, CD3 on secreting Treg, CD38 on IgD − CD38dim, CD8dim %T cell, Naive DN (CD4 − CD8 −) AC) showed a positive correlation with the onset of in situ of cervix uteri, with IVW OR(95% CI, P) of 1.059 (1.016 to 1.103, 0.006), 1.063 (1.005 to 1.124, 0.032), 1.150 (1.063 to 1.245, 0.001), 1.055 (1.004 to 1.108, 0.034), 1.09 (1.009 to 1.178, 0.028), 1.064 (1.015 to 1.115, 0.01), 1.089 (1.000 to 1.185, 0.049), 1.085 (1.025 to 1.148, 0.005), 1.050 (1.006 to 1.095, 0.027), 1.117 (1.014 to 1.231, 0.025), and 1.118 (1.011 to 1.235, 0.029). The alignment between IVW and other four MR techniques was evident in most MR analyses (Supplementary Fig. 3). Notably, the p value of the MR-PRESSO test for HLA DR on B cell was less than 0.001, indicating the presence of horizontal heterogeneity between exposure factors and outcome variables. Furthermore, reverse MR analysis revealed evidence of a causal relationship between in situ of the cervix uteri and HLA DR on B cells. As a result, HLA DR on B cells was eliminated from consideration. Collectively, 11 types of immune traits were demonstrated to exert a causal effect on carcinoma in situ of the cervix uteri.

### Causal effect of immune traits on malignant CC

**Figure 4:**
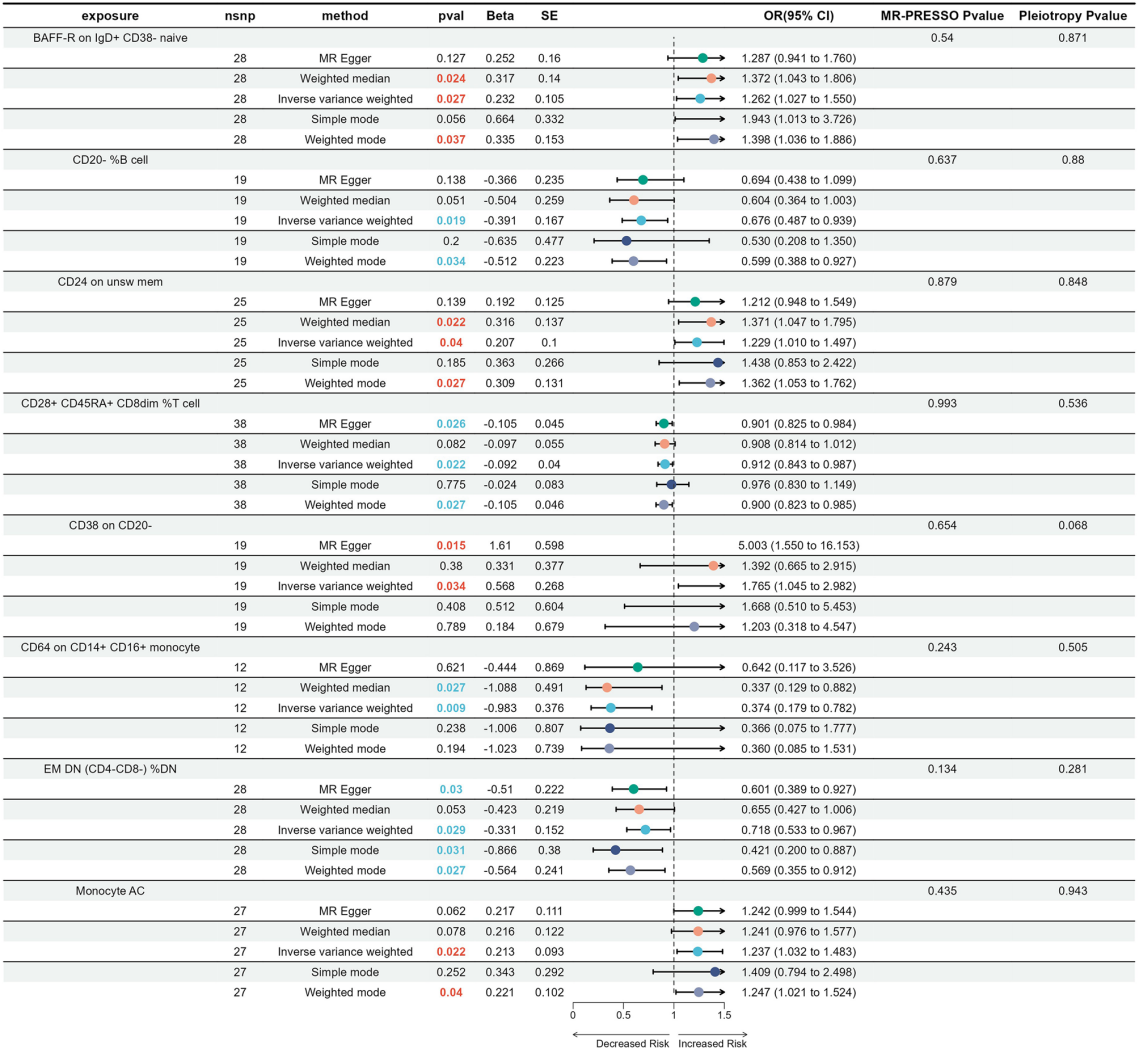
Forest plot showing the causal effects of immune cell traits and adenocarcinomas of cervix.

To investigate the causal effect of immune traits on malignant CC, a two-sample MR analysis was performed using GWAS data of adenocarcinomas of cervix, SCNCC, and malignant neoplasm of cervix uteri as outcome variables. For adenocarcinomas of cervix, eight immune traits belonging to three cell types (B cell, T cell, and monocyte) were identified (Fig. 4). Among the identified cell subtypes, four types of immune traits were positively correlated with adenocarcinomas of cervix, while the other four immunophenotypes were negatively correlated with the disease. It is worth noting that different subtypes of the same class of cells surprisingly play varying roles in the onset of diseases. B cell subsets BAFF-R on IgD+ CD38− naive, CD24 on unsw mem, and CD38 on CD20− were positively associated with the occurrence of adenocarcinomas of cervix, whereas CD20− %B cells exhibited a protective effect against the disease. Similarly, Monocyte AC showed a positive correlation with adenocarcinomas of cervix, while CD64 on CD14+ CD16+ monocytes had a negative causal effect on adenocarcinomas of the cervix. This result indicates that in-depth study of cell typing is of crucial significance for understanding the occurrence and development of CC. There was no evidence of a causal relationship between adenocarcinomas of cervix and the eight immune traits in the reverse MR analysis. Supplementary Tables 3, 4 and 5 provided a comprehensive overview of the detailed findings. In summary, a causal relationship between adenocarcinomas of the cervix and the eight immune traits was identified.

**Figure 5:**
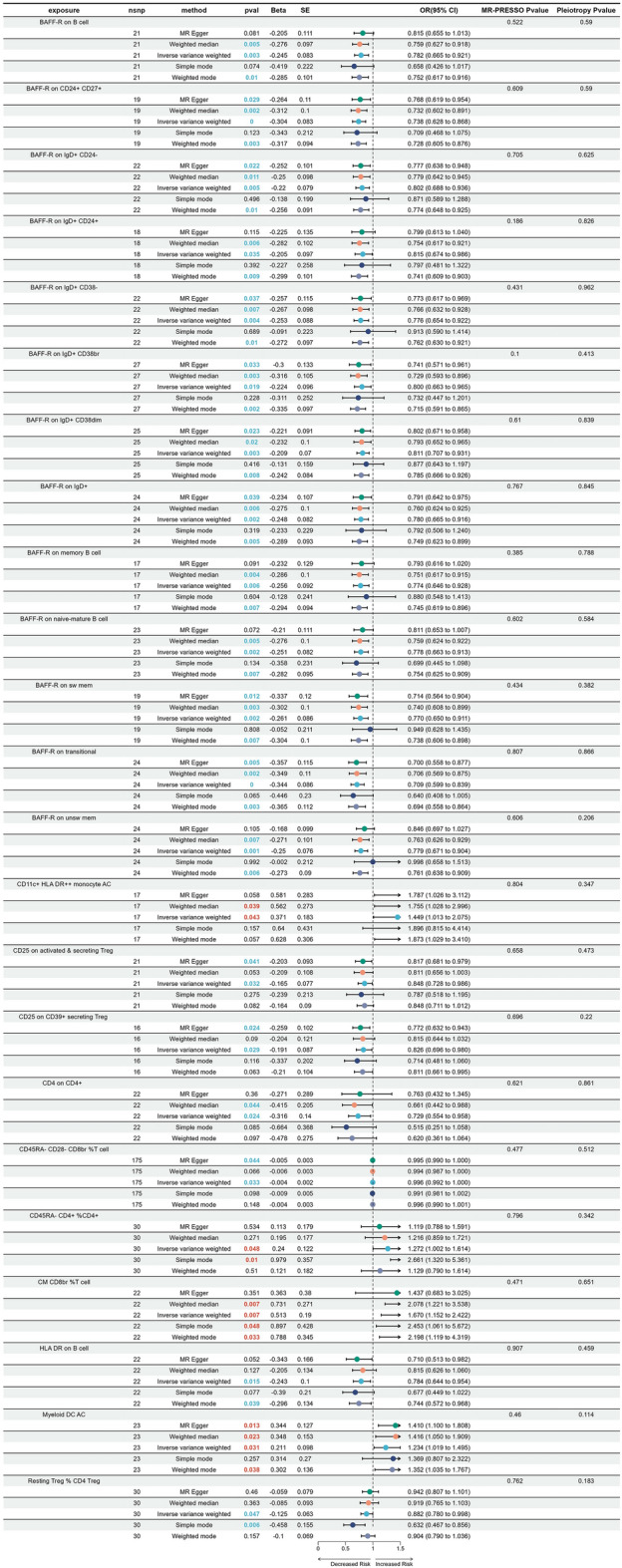
Forest plot revealed the causal effects of immune cell traits and squamous cell neoplasms and carcinoma of cervix.

Considering SCNCC, a total of 23 immune traits were found to be associated with the disease (Fig. 5). Most of these immunophenotypes were identified as protective factors, while only four showed a positive correlation with the onset of the disease. Out of the 23 identified subtypes of immune cells, 14 are B cells, and 13 of these cells express BAFF-R (B-cell activating factor receptor). Therefore, the expression of BAFF-R may have a protective effect on the development of squamous CC. Different subtypes of T cells exert different functions, but regulatory T cells exhibit a protective effect. Like adenocarcinomas of cervix and non-neoplastic diseases, monocytes (CD11c+ HLA DR++ monocyte AC) showed a positive correlation trend with disease progression. In the reverse MR analysis, no evidence was found to suggest a causal relationship between SCNCC and the 23 immune traits. Supplementary Tables 6, 7 and 8 presented a detailed summary of the findings.

**Figure 6:**
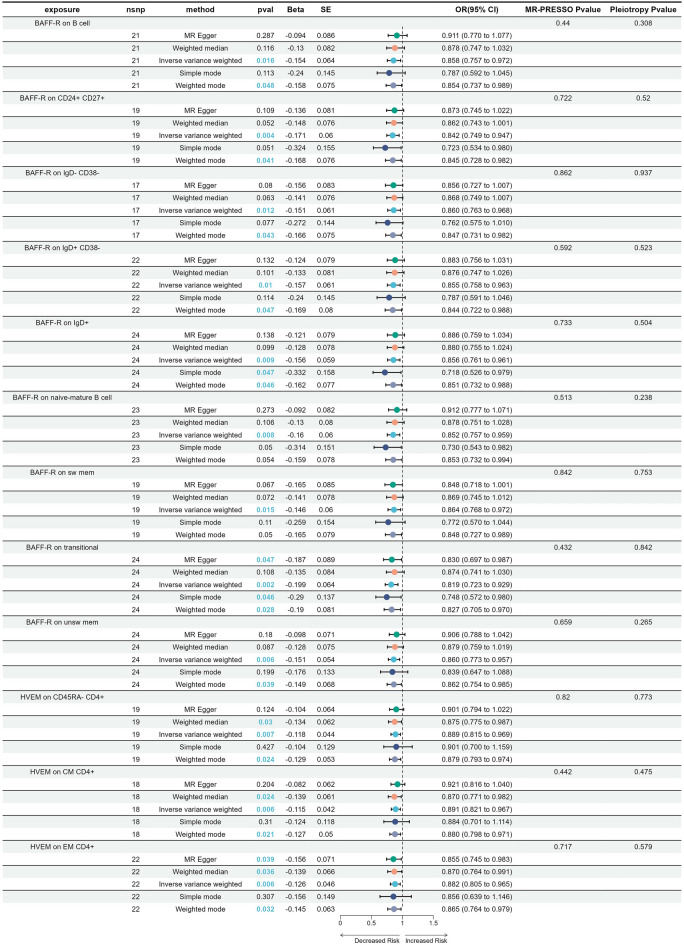
Forest plot to depict the causal effects of immune cell traits and malignant neoplasm of cervix uteri.

In cases of malignant neoplasm of the cervix uteri, a total of 12 immune traits were screened (Fig. 6). Supplementary Tables 9, 10 and 11 demonstrated a detailed summary of the findings. Nine were B cell and three were T cell, all of which were found to have a negative association with the onset of the disease. Similar to SCNCC, the B cell was BAFF-R positive. Among these eight B cell subtypes, except for BAFF-R on IgD− CD38−, the others were identified as disease-relevant factors in SCNCC. Furthermore, BAFF-R on IgD + CD38− and BAFF-R on IgD− CD38− only differ in IgD expression, but both have been identified as having a causal relationship with malignant neoplasm of the cervix uteri. This result further emphasizes the importance of BAFF-R in the development of malignant CC. The three T cells expressed HVEM (Herpesvirus entry mediator), with HVEM on CM CD4+ and HVEM on EM CD4+ belonging to HVEM on CD45RA−CD4+ . No evidence was found in the reverse MR analysis to suggest a causal relationship between malignant neoplasm of the cervix uteri and the 12 immune traits. Collectively, a causal effect of immune traits was identified in three types of malignant CC, namely adenocarcinomas of cervix, SCNCC, and malignant neoplasm of cervix uteri.

## Discussion

TME plays a crucial role in cancer initiation and progression, with T cells, B cells, NK cells, stromal cells, NKT cells, and fibroblasts being the main components of TME. Therefore, research on the composition of immune cells in the TME is vital for understanding tumor onset and development. In this study, four types of immune traits were found to be associated with the onset of inflammatory diseases of the cervix: B cells, T cells, cDCs, and monocytes. Specifically, CCR2 on monocytes and CD11c on monocytes were identified as potential factors increasing the risk of inflammatory diseases of the cervix. These traits characterize classical monocytes known for their proinflammatory nature and high production of various inflammatory factors. Additionally, CD11c, a member of the integrin family, facilitates cell migration during inflammatory responses. We also found that CCR2 on CD14−CD16− (Monocyte panel) was linked to a higher likelihood of developing cervical disorders. CCR2 (C–C motif chemokine receptor 2) is expressed on classical monocytes and interacts with CCL2 to facilitate the migration of monocytes^26,27^. Classical monocytes exhibit a proinflammatory nature and generate the highest amounts of CCL2, granulocyte colony-stimulating factor (CSF), and IL-6/10^28^. Additionally, they release high levels of reactive oxygen species (ROS) and show the highest production of CCL2/3 and IL-8/10 when stimulated with lipopolysaccharide (LPS)^29^. During inflammatory responses, cell migration is facilitated by CD11c, a member of the integrin family, through cell–cell adhesion^30^. With other noninflammatory disorders of the cervix uteri, CCR2 on CD14−CD16− (monocyte panel) was linked to a higher likelihood of developing cervical disorders. Combining our analysis results with the experimental results of previous researchers, we believe that classic monocytes may play an important role in the non-neoplastic conditions of CC through their pro-inflammatory effects. In the other immune cell phenotypes we identified, aside from CD45 on NK cells, all others were specific immune cells, indicating a potential role of acquired immunity in inhibiting the progression of diseases.

For adenocarcinomas of cervix, the absolute count of monocytes was found to be positively correlated with the disease. However, the presence of CD64 on CD14+ CD16+ monocytes was identified as having a negative causal effect. In humans, three primary subsets of monocytes have been recognized: classical (CD14+ CD16−), intermediate (CD14+ CD16+), and nonclassical (CD14−CD16+−)^31^. These subsets exhibit functional variances, including their responses to inflammation and ability to migrate^32^. Intermediate monocytes exhibit elevated levels of HLA-DR, CD80, CD86, and TNFR1, suggesting their involvement in both antigen presentation and inflammatory functions^33^. In addition, intermediate monocytes demonstrate genes that oversee chemotaxis and angiogenesis (AIF1 and TIE2) as well as phagocytosis and tissue repair (TGFB and CD93)^34^. This suggests that intermediate monocytes, despite being considered inflammatory, are also likely participating in anti-inflammatory processes^35^. We believe that CD64 on CD14+ CD16+ monocytes plays an important anti-inflammatory role in the pathogenesis of adenocarcinomas of the cervix, showing a negative correlation with disease progression. The percentage of CD20- in B cell was negatively associated with adenocarcinomas of cervix, however, CD38 on CD20- (B cell) was a risk factor for disease progression. Consequently, further research is necessary to deepen our understanding of the relationship between the classification of immune cells and adenocarcinomas of the cervix.

Compared with other types of cervical diseases, SCNCC present with more disease-related factors. Specifically, CD11c+ HLA DR++ monocyte AC was positively correlated with disease progression in these conditions. Almost all of the 14 identified B cell types that offer protective effects against the disease express BAFF-R. BAFF, also known as CD257, TNFSF13B, and BLyS, belongs to the tumor necrosis factor (TNF) family. It binds specifically to BAFF-R (TNFRSF13C) and plays a critical role in regulating B-cell functions and autoimmune diseases like systemic lupus erythematosus (SLE) and blood cancers^36^. B-cell subsets exhibit varying levels of expression of BAFF system receptors^37^. BAFF promotes B-cell growth and survival both in vitro and in vivo by enhancing protein levels and activating genes linked to glucose uptake and glycolytic metabolism, leading to B-cell hyperactivity in response to mitogenic stimulation during inflammation^38^. Based on our analysis and previous studies, we believe that the expression of BAFF-R in B cells plays a crucial protective role in the progress of SCNCC. Similarly, BAFF-R positive B cells are beneficial in the advancement of malignant neoplasm of the cervix uteri, indicating the significance of BAFF-R.

In pre-cancerous lesions and malignant cervical cancer, acquired immune cells typically play a protective role in the development of the disease. However, in the in situ of cervix uteri, T cells and B cells have been identified as being associated with the progression of the disease, with all cells playing a promotive role in its development. This analysis result has significant contradictions with our perception. There was a widespread agreement on the important role of effector T cells in antitumour responses^39^. Tumors can be categorized based on how cytotoxic immune cells are dispersed within the tumor microenvironment (TME) into immune-inflamed, immune-excluded, and immune-desert phenotypes. Based on the dispersion of cytotoxic immune cells within the TME, a tumor can be classified into one of three primary immunophenotypes: immune-inflamed, immune-excluded, and immune-desert phenotypes^40^. Immune-inflamed tumors, also known as "hot tumors", are characterized by high levels of T-cell infiltration, increased interferon-y (IFN-y) signaling, and a high tumor mutational burden (TMB)^41^. Conversely, immune-excluded and immune-desert tumors are commonly known as 'cold tumors'^42,43^. In immune-excluded tumors, CD8+ T lymphocytes are limited to the edges of the tumor invasion and do not effectively penetrate the tumor^43^. Therefore, we suspect that in the case of cervix uteri, it may exhibit characteristics of immune-excluded tumors. Despite having a large number of T cells, they do not infiltrate the tumor tissues effectively, indicating a positive correlation between the T cell phenotype and the disease.

In conclusion, this study involved the analysis of GWAS data for 731 immune traits and six GWAS data sets for CC and cervical non-cancer cases. Two-sample MR analysis was conducted to determine the causal role of immune cells in the onset of CC. Our findings revealed a positive correlation between classic monocytes and various cervical diseases. Additionally, we observed that B cells expressing BAFF-R have an inhibitory effect on malignant CC, specifically SCNCC. The results of our analysis provide valuable insights into the pathogenesis of cervical diseases and offer potential implications for the clinical treatment of cervical cancer through immune cell subtyping.

## Conclusion

In conclusion, this study involved the analysis of GWAS data for 731 immune traits and six GWAS data sets for CC and non-cancer cases. Two-sample MR analysis was conducted to determine the causal role of immune cells in the onset of CC. Our findings revealed a positive correlation between classic monocytes and various cervical diseases. Additionally, we observed that B cells expressing BAFF-R have a inhibitory effect on malignant cervical cancer, specifically SCNCC. Our analysis yields valuable insights into the pathogenesis of cervical diseases, revealing a correlation between local immune status and cervical lesions through analysis integrated with GWAS.

### Supplementary Information

Supplementary Figure 1.

Supplementary Figure 2.

Supplementary Figure 3.

Supplementary Table 1.

Supplementary Table 2.

Supplementary Table 3.

Supplementary Table 4.

Supplementary Table 5.

Supplementary Table 6.

Supplementary Table 7.

Supplementary Table 8.

Supplementary Table 9.

Supplementary Table 10.

Supplementary Table 11.

## Data Availability

The data utilized in this study were obtained from the GWAS platform (https://gwas.mrcieu.ac.uk/) and the FinnGen Research (https://risteys.finregistry.fi/), both of which are freely accessible.
