# Supplementary figures and images for: Causal role of immune cells on cervical cancer onset revealed by two-sample Mendelian randomization study
Source: Sci Rep. 2024 Jun 28;14:14890. doi: 10.1038/s41598-024-65957-7 (PMC11211447; doi:10.1038/s41598-024-65957-7)

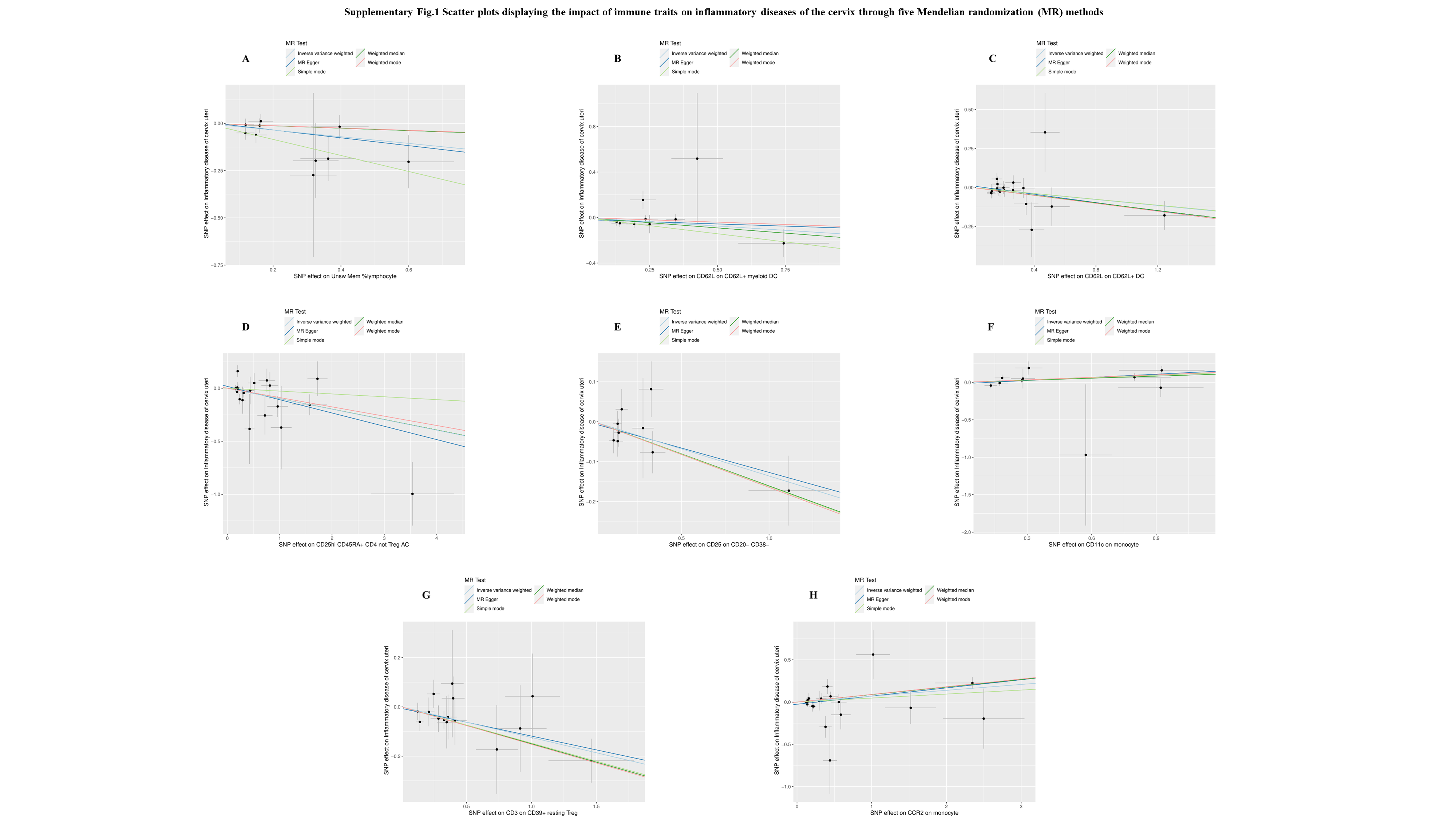

Supplement: Supplementary file 1 — Supplementary Figure 1. [file 41598_2024_65957_MOESM1_ESM.tif]

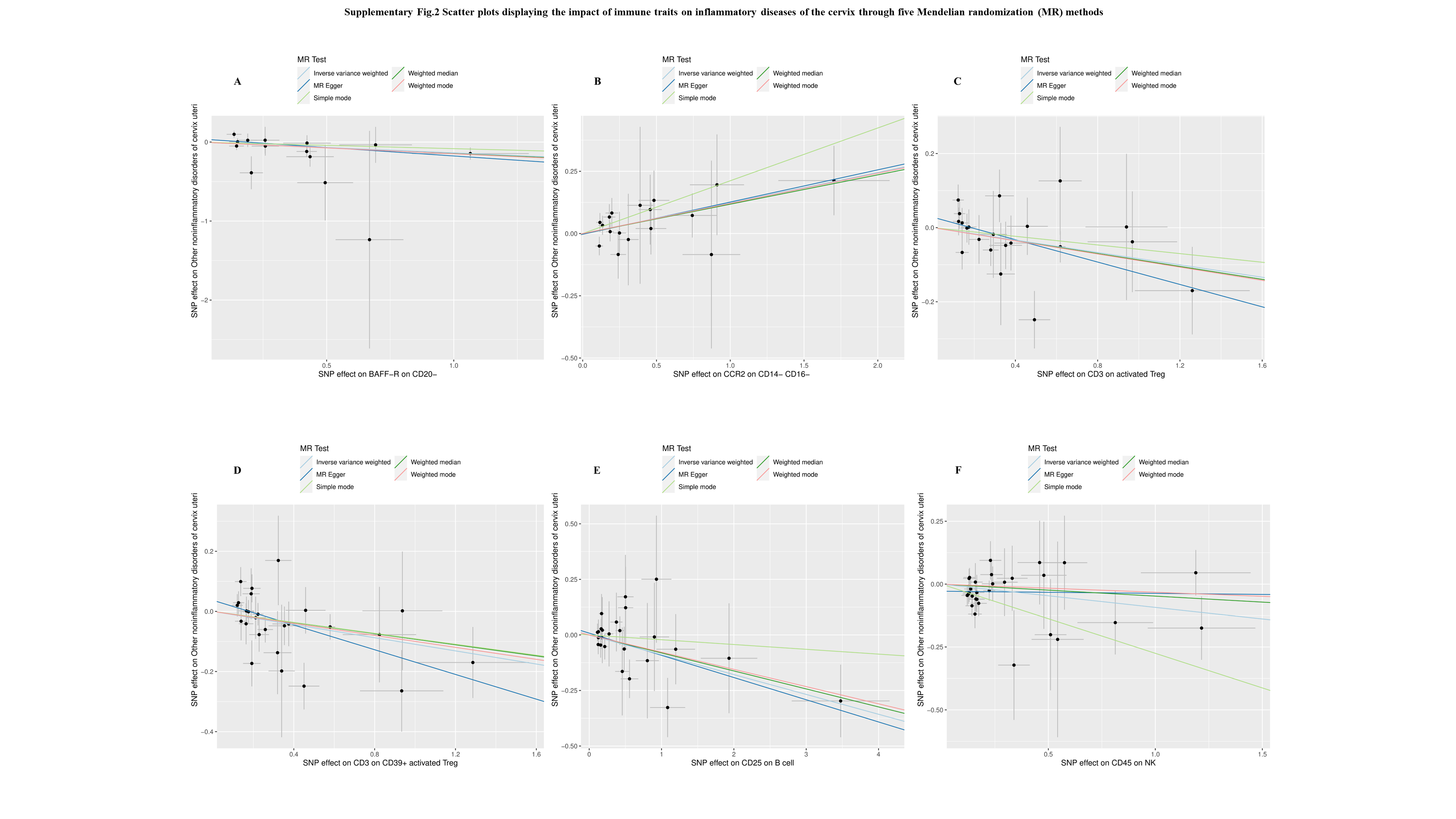

Supplement: Supplementary file 2 — Supplementary Figure 2. [file 41598_2024_65957_MOESM2_ESM.tif]

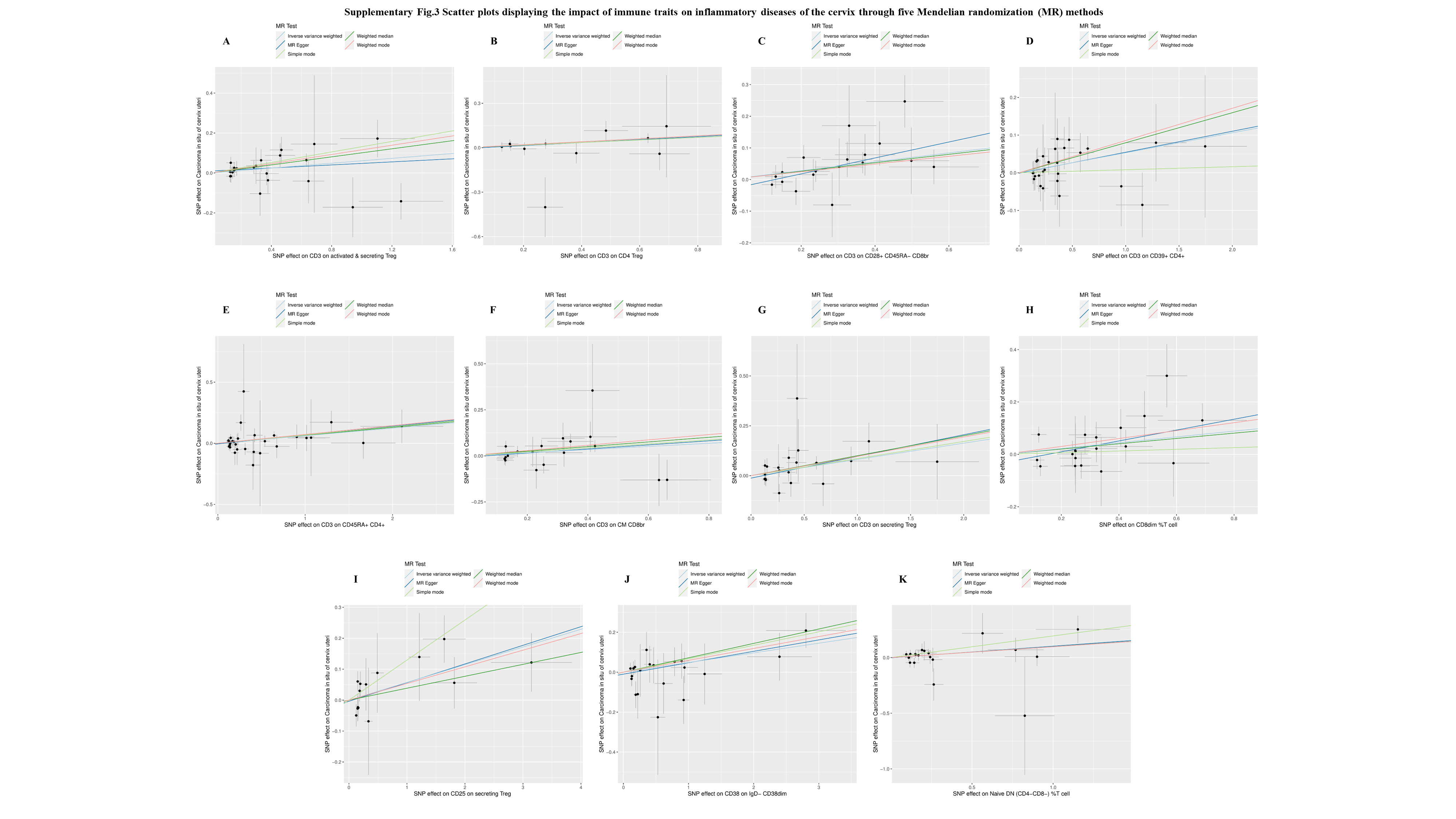

Supplement: Supplementary file 3 — Supplementary Figure 3. [file 41598_2024_65957_MOESM3_ESM.tif]
