# Supplementary material for: Causal role of immune cells on cervical cancer onset revealed by two-sample Mendelian randomization study
Source: Sci Rep. 2024 Jun 28;14:14890. doi: 10.1038/s41598-024-65957-7 (PMC11211447; doi:10.1038/s41598-024-65957-7)
Supplement: Supplementary file 4 — Supplementary Table 1. [file 41598_2024_65957_MOESM4_ESM.docx]

**Supplementary table 1 The detailed information of GWAS data for cervical cancer (CC) and cervical non-neoplastic conditions used in this study. The information could be found by visiting the following website: https://icd.who.int/browse10/2016/en.**

| **Type** | ***Incl*** | ***Excl*** | **Website** | **International Statistical Classification of Diseases and Related Health Problems 10th Revision (ICD-10)-WHO Version for ;2016** |
| --- | --- | --- | --- | --- |
| Inflammatory disease of cervix uteri | Cervicitis (with or without erosion or ectropion) | erosion and ectropion of cervix without cervicitis | https://risteys.finregistry.fi/endpoints/N14_INFCERVIX | https://icd.who.int/browse10/2016/en#/N72 |
|  | Endocervicitis (with or without erosion or ectropion) |  |  |  |
|  | Exocervicitis (with or without erosion or ectropion) |  |  |  |
| Other noninflammatory disorders of cervix uteri | N88.0 Leukoplakia of cervix uteri | inflammatory disease of cervix (N72),  polyp of cervix (N84.1) | https://risteys.finregistry.fi/endpoints/N14_OTHNONINFCERVIX | https://icd.who.int/browse10/2016/en#/N88 |
|  | N88.1Old laceration of cervix uteri Adhesions of cervixExcl.:current obstetric trauma (O71.3) |  |  |  |
|  | N88.2Stricture and stenosis of cervix uteri Excl.:complicating labour (O65.5) |  |  |  |
|  | N88.3Incompetence of cervix uteri Investigation and management of (suspected) cervical incompetence in a nonpregnant womanExcl.:affecting fetus or newborn (P01.0)complicating pregnancy (O34.3) |  |  |  |
|  | N88.4Hypertrophic elongation of cervix uteri |  |  |  |
|  | N88.8Other specified noninflammatory disorders of cervix uteri  Excl.:current obstetric trauma (O71.3) |  |  |  |
|  | N88.9Noninflammatory disorder of cervix uteri, unspecified |  |  |  |
| Carcinoma in situ of cervix uteri (controls excluding all cancers) | D06.0  Endocervix | melanoma in situ of cervix (D03.5) severe dysplasia of cervix NOS (N87.2) | https://risteys.finregistry.fi/endpoints/CD2_INSITU_CERVIX_UTERI_EXALLC | https://icd.who.int/browse10/2016/en#/D06 |
|  | D06.1  Exocervix |  |  |  |
|  | D06.7  Other parts of cervix |  |  |  |
|  | D06.9  Cervix, unspecified |  |  |  |
| Adenocarcinomas of cervix (controls excluding all cancers) |  |  | https://risteys.finregistry.fi/endpoints/C3_CERVIX_ADENO_EXALLC |  |
| Squamous cell neoplasms and carcinoma of cervix (controls excluding all cancers) |  |  | https://risteys.finregistry.fi/endpoints/C3_CERVIX_SQUAM |  |
| Malignant neoplasm of cervix uteri (controls excluding all cancers) | C53.0  Endocervix |  | https://risteys.finregistry.fi/endpoints/C3_CERVIX_UTERI | https://icd.who.int/browse10/2016/en#/C53 |
|  | C53.1  Exocervix |  |  |  |
|  | C53.8  Overlapping lesion of cervix uteri [See note 5 at the beginning of this chapter] C |  |  |  |
|  | 53.9  Cervix uteri, unspecified |  |  |  |
